# Outcomes following balloon tibioplasty versus conventional osteosynthesis techniques for Schatzker type III tibial plateau fractures: a systematic review

**DOI:** 10.1186/s13018-022-02973-1

**Published:** 2022-04-07

**Authors:** Andrew Blankenship, Amy Singleton, Logan Hiatt, Kirk W. Evanson, Seth Phillips, Richard Miller

**Affiliations:** 1 Department of Orthopedics, Mercy Health St. Vincent Medical Center, 2409 Cherry St, Toledo, OH 43608 USA; 2 Superior Medical Experts, 1425 Minnehaha Ave E, P.O. Box 600545, St. Paul, MN 55106 USA

**Keywords:** Tibia, Tibial plateau, Balloon tibioplasty, Schatzker, Internal fracture fixation, Articular range of motion

## Abstract

**Introduction:**

Schatzker type III fractures of the tibial plateau require elevation of the depressed portions to regain articular congruity. Balloon tibioplasty has been used as an alternative to conventional metal instruments for elevation of the lateral tibial plateau. This study compared functional outcomes following balloon tibioplasty or conventional osteosynthesis techniques in patients with type III fractures of the tibial plateau.

**Materials and methods:**

A systematic literature search was performed using PubMed, EMBASE, and Cochrane Library to identify studies published through March 29, 2021, pertaining to balloon tibioplasty or conventional osteosynthesis techniques for type III fractures. Non-human studies, opinion or editorial pieces, systematic reviews, case series (< 5 patients), and articles published in a non-English language were excluded. Primary outcomes were Rasmussen clinical score, range of motion, and Knee Society Score (KSS). A Joanna Briggs Institute (JBI) risk of bias assessment was performed for all studies.

**Results:**

A total of 95 studies were identified, with 10 studies (and 132 total patients) meeting inclusion criteria: 1 study focused on balloon tibioplasty, 8 studies reported outcomes following conventional osteosynthesis, and 1 study compared outcomes of the two techniques. Mean follow-up times varied widely, from 4 to 76.3 months. Where reported, balloon tibioplasty resulted in good to excellent functional outcomes as indicated by Rasmussen clinical scores (mean 28.3 in a case series; mean 28.9 in a randomized controlled trial) and range of motion (≥ 140° in both studies) 1–2 years following surgery. KSS was not reported consistently enough for comparison. Studies ranged from low to high risk of bias according to the JBI assessment.

**Conclusions:**

Balloon tibioplasty can lead to excellent functional outcomes in patients with depression fractures of the lateral tibial plateau. More research is needed to directly compare outcomes following treatment with balloon tibioplasty or conventional osteosynthesis techniques.

**Supplementary Information:**

The online version contains supplementary material available at 10.1186/s13018-022-02973-1.

## Introduction

A tibial plateau fracture is a common intra-articular injury that typically results from a low energy mechanism, accounting for 1% of all fractures and 8% of fractures in the elderly [1, 2]. Up to 70% of patients sustaining a tibial plateau fracture are older than 50 years [3]. The most common categorization system, the Schatzker system, was first published in the 1970s and categorizes tibial plateau fractures by the location of the fracture plane, from lateral to medial, based on a two-dimensional representation of the tibia [4]. With the advent of computed tomography (CT) and three-dimensional (3D) reconstruction, Schatzker redefined the classification system in 2018 to include anterior and posterior modifiers in order to better describe the fracture plane [5]. Here, we specifically discuss Schatzker type III fractures, which involve depression of the lateral tibial metaphysis and constitute up to 36% of all tibial plateau fractures [6].

When a patient presents with a tibial plateau fracture, the main goals of intervention are reestablishing mechanical alignment, stability, and articular congruity to minimize posttraumatic arthritis [1, 7], but optimal fixation methods remain controversial. Methods of surgical treatment for tibial plateau fracture vary from open reduction and internal fixation (ORIF) to acute total knee replacements. Locking plates are often used for internal fixation, with bone grafts, cancellous chips, or bone cement used to fill the metaphyseal space [8]. For type III fractures, elevation of the depressed portion of the metaphysis is crucial to regaining articular congruity.

Tibial plateau reduction and fixation is a challenging procedure due to vulnerable nervous, vascular, and connective tissue; every millimeter of articular depression can result in up to 2° of limb malalignment, with surgical fixation indicated for malalignments in the range of 5–10° [9]. Additionally, because type III fractures are more common in older individuals, the use of metal instruments, such as a tamp, for reduction in these patients can lead to iatrogenic damage of the articular surface and possibly lead to increased levels of post-traumatic arthritis [10, 11].

The use of a kyphoplasty balloon (balloon tibioplasty) for elevation and reduction of depressed metaphyseal portions is a minimally invasive approach growing in popularity. The balloon forms a symmetric cavity that can then be filled with bone filler to improve subchondral support [12]. Traditionally used in vertebral compression fractures to assist in restoring vertebral body height, kyphoplasty balloons are made of a soft material with a large surface area and easy maneuverability, which makes them ideal for reduction of type III fractures [13]. Additionally, balloon osteoplasty has already been established as a safe technique for reduction of compression fractures of the hip [14], foot [13, 15], and wrist [16].

Functional outcomes after balloon tibioplasty for type III fractures are poorly understood. A recent systematic review from Sinha and Maffulli demonstrated favorable outcomes with balloon tibioplasty, but did not compare this technique to other, more conventional surgical techniques with plates and screws [7]. The present study compares functional outcomes following balloon tibioplasty reduction versus conventional osteosynthesis techniques for type III tibial plateau fractures.

## Methods

### Literature search

A PRISMA-compliant systematic literature search was conducted using PubMed, Cochrane Library, and EMBASE databases for articles published up to March 29, 2021 [17]. No ethics approval was required for this systematic review of the literature. The search terms used for each database are presented in Table 1. Studies that presented functional outcomes of interest, including Rasmussen score and range of motion (ROM), in patients with type III fractures following balloon tibioplasty or conventional, non-balloon surgical techniques were included; otherwise, there were no specific inclusion/exclusion criteria around surgical techniques. The following article types were excluded: non-human studies, opinion or editorial pieces, systematic reviews, case series (< 5 patients), and articles published in a non-English language.

**Table 1:** Search strategy by database

	Search strings	Databases
1	(Schatzker AND type AND III AND (“tibia”[MeSH Terms] OR “tibia”[All Fields] OR “tibial”[AllFields]) AND plateau[All Fields] AND (“fractures, bone”[MeSH Terms] OR (“fractures”[All Fields] AND “bone”[All Fields]) OR “bone fractures”[All Fields] OR “fractures”[All Fields]))	PubMed
2	(Schatzker AND type AND III AND (“tibia” OR “tibia”[All Fields] OR “tibial”[AllFields]) AND plateau[All Fields] AND (“fractures, bone” OR (“fractures”[All Fields] AND “bone”[All Fields]) OR “bone fractures”[All Fields] OR “fractures”[All Fields]))	EMBASE
3	(Schatzker AND type AND III AND (“tibia” OR “tibia”[All Fields] OR “tibial”[AllFields]) AND plateau[All Fields] AND (“fractures, bone” OR (“fractures”[All Fields] AND “bone”[All Fields]) OR “bone fractures”[All Fields] OR “fractures”[All Fields]))	Cochrane

### Data variables extracted

Variables extracted from the articles included number of subjects, type of surgical reduction and fixation method used, length of subject follow-up, pain levels, Rasmussen scores, Knee Society Scores (KSS), and postoperative ROM values. The Rasmussen scoring system assesses five different domains (pain, walking capacity, extension lag, range of motion, and stability), with scores of 28–36 indicating “excellent,” 20–27 indicating “good,” 10–19 indicating “fair,” and 6–9 indicating “poor” evaluations [3]. Due to limited data, no inferential statistics were used. Data are reported as frequencies, counts, percentages, and ranges.

### Risk of bias assessment

The Joanna Briggs Institute (JBI) risk of bias form was used to assess all studies for risk of bias [18]. The form is tailored according to study design (case series, cohort studies, etc.) and questions span 10–12 different domains of potential sources of bias, with possible responses of “yes,” “no,” “unclear,” or “not applicable.” Responses are synthesized to form an overall judgment of low, moderate, or high risk of bias, along with recommendations for study inclusion or exclusion. Two independent reviewers performed the risk of bias assessment for each study in this systematic review, with a third reviewer adjudicating differences.

## Results

### Search results

**Fig. 1:**
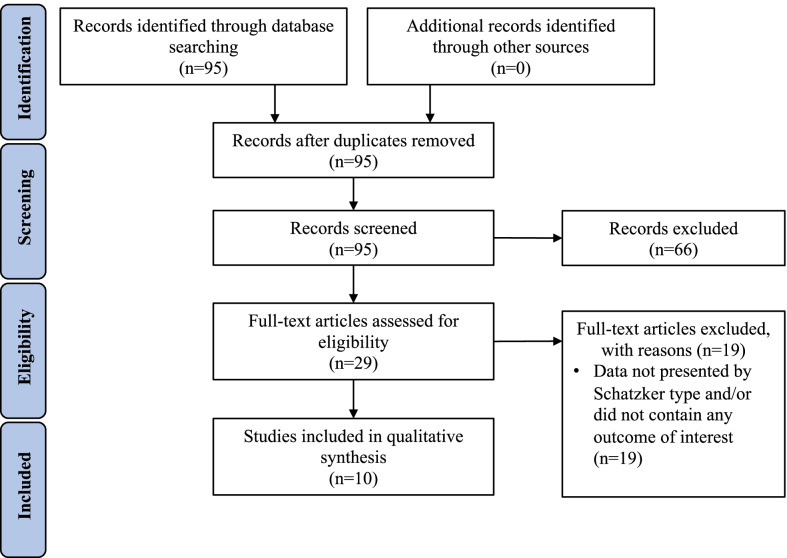
PRISMA diagram of search records and included studies. (Descriptive caption: flowchart indicates 95 records were identified through database searching, 0 additional records were identified through other sources, 95 records remained after duplicates were removed, 95 total records were screened, 66 records were excluded based on title/abstract, 29 full-text articles were assessed for eligibility, 19 full-text articles were excluded for not presenting data by Schatzker type and/or not containing any outcome of interest, 10 studies were included in qualitative synthesis)

A total of 95 articles were screened. After review of titles/abstracts, 29 articles were selected for full-text review, with a total of 10 articles (and 132 total patients) ultimately meeting inclusion criteria (Fig. 1). Studies were published from 1997 to 2019 [19, 20]. One article (Cuzzocrea et al.) described outcomes following balloon tibioplasty [21], 8 articles described conventional, non-balloon-assisted surgical techniques [19, 20, 22–27], and 1 article (Doria et al.) compared outcomes of balloon tibioplasty versus conventional techniques [28]. Mean follow-up times varied widely, from 4 to 76.3 months [19, 22]; mean age and age distributions were comparable between groups. Nine articles described one or more outcomes of interest in patients with only type III fractures [19–27]. An additional article compared outcomes following balloon tibioplasty or conventional osteosynthesis techniques in 28 patients with lateral depression fractures of the tibia [28], of which 25 patients (89.3%) had experienced type III fractures and 3 (10.7%) patients had experienced type II fractures (lateral split with depression). Study-level characteristics are shown in Table 2.

**Table 2:** Summary data of balloon and non-balloon reduction of Schatzker type III fractures

Author	N	Surgical details	F/U** (mo.)	Age (years)	Rasmussen	KSS	ROM (°)
*Balloon tibioplasty*							
Cuzzocrea et al. [21]	6	ARIF; reduction with kyphoplasty balloon, calcium phosphate filler	24	46 *(36–55)*	28.3 ± 0.8	–	140 [140–140]
Doria et al. [28]	14	Reduction with inflatable bone tamp; calcium phosphate filler, 13 pts had no fixation, 1 pt had plate inserted due to previous surgery	14	67 *(23*–*74)*	28.9	–	140 [140–140]
*Conventional*							
Bansal et al. [25]	6	ORIF, bovine cancellous graft, plating, or cannulated screw fixation	12*	74 *(63*–*86)*	–	–	90 [86.25–90]
Dall’Oca et al. [23]	26	ARIF, cannulated screws or plates and screws	73*	51 *(13–77)*	28.62	–	–
	18	ORIF, cannulated screws or plates and screws	27*		28	–	–
Doria et al. [28]	14	Traditional reduction with metal tamp, bone substitute (calcium phosphate); fixation with cannulated screws (4 pts) and buttress plates (10 pts)	14	65 *(21–73)*	26.1	–	140 [125–140]
Haq et al. [26]	10	ORIF, fixation could involve screws and plates	6*	N/A	20	–	120 [90–120]
Jagdev et al. [22]	7	Fixation could include cannulated screws and/or plates	76.32* [42–130]	41 *(20–73)*	–	^†^80–100 **(7)**	–
Lasanianos et al. [27]	6	ORIF, reduced by direct elevation, freeze-dried cancellous allograft chips used to fill void, plate support	13*	53 *(20–73)*	18.0 ± 0.6	–	130 [130–133.75]
Raza et al. [24]	11	Minimally invasive plate osteosynthesis, cancellous autologous bone graft filler, plates, cancellous or cannulated screws for some patients	12	40 ± 14 *(19–75)*	24.5 ± 3.3	–	120 [120–120]
Touliatos et al. [19]	5	ORIF, autologous cancellous bone graft filler, could include plate and screw support	4*	N/A	–	80–100 **(5)**	–
Zawam et al. [20]	9	ARIF, reduction with impactor, iliac crest bone graft, fixed with 2 cannulated screws and washers	14* [11–18]	39 *(19–55)*	27–30 **(7)**; 20–26 **(2)**	–	–

Data are expressed as mean, mean ± standard deviation, mean *(range),* median[interquartile range], or as a given value range (**number of patients in that range**)

^*^Includes only Schatzker type III fractures

^**^Follow-up is expressed as a specific time point or as mean follow-up of all Schatzker types in the study

^†^American Knee Society Score

*KSS* Knee Society Score, *F/U* follow-up, *ROM* range of motion, *N/A* not available, *ORIF* open reduction internal fixation, *ARIF* arthroscopic reduction internal fixation, *pt(s)* patient(s), *mo* months

### Risk of bias assessment

Of the ten included studies, four (4/10, 40.0%) were considered low risk of bias, four (4/10, 40.0%) were considered moderate risk of bias, and two (2/10, 20.0%) were considered to have high risk of bias. Risk of bias did not correlate with study design type; the one cohort study included was deemed high risk of bias [23], and the one randomized control trial was deemed moderate risk of bias [28]; the remaining eight studies were considered case series. All studies were ultimately recommended for inclusion in the systematic review. Full details of the risk of bias assessment are shown in Additional file 1.

### Rasmussen and Knee Society Scores

The Rasmussen clinical assessment was the most common functional outcome, with 70.0% (7/10) of studies reporting for 86.4% (114/132) of included patients (Table 2). In patients that received balloon tibioplasty, Cuzzocrea et al. [21] reported good Rasmussen scores in patients at 6 months following surgery (mean 26.3, n = 6), with excellent function achieved at 12 (mean 28.3) and 24 months (mean 28.8). Doria et al. [28] reported excellent Rasmussen scores (mean 28.9, n = 14) approximately 14 months following balloon tibioplasty. This same study noted slightly lower Rasmussen scores (mean 26.1, n = 14) at same follow-up for patients receiving conventional reduction. Follow-up times and Rasmussen scores varied widely across other studies that examined conventional surgical techniques for type III fractures. Fair to excellent Rasmussen scores were reported for conventional surgical techniques at an intermediate follow-up of approximately 12–14 months [20, 24, 27]. For the conventional surgical technique group, Dall’Oca et al. [23] reported the highest mean Rasmussen scores (27–30) at 2–6 years follow-up.

KSS were recorded in only two studies; one utilized ORIF and one utilized a conventional, non-balloon surgical technique for reduction of type III fractures. Excellent results were observed at four-month follow-up in 100.0% (5/5) patients that received ORIF [19], and in 100.0% (7/7) patients that received non-balloon reduction at a mean of 76.3 months follow-up [22].

### Postoperative range of motion

Of the patients that received balloon tibioplasty for type III fractures, 95.0% (19/20) experienced full ROM (≥ 140°) at 1- or 2-year follow-up [21, 28]. In the randomized trial by Doria et al. [28], 92.9% (13/14) of patients receiving balloon tibioplasty experienced full ROM at 1-year (mean 14 months) follow-up, while 71.4% (10/14) of patients receiving metal tamp reduction experienced full ROM. Of patients with type III fractures receiving conventional osteosynthesis, median values ranged from ≥ 90° to ≥ 130° at final follow-up (approximately 6–12 months) [25–27]. More details are given in Table 2.

### Postoperative pain

Pain levels were noted across several studies either independently or as part of Rasmussen scores, which distinguishes between pain temporality, severity, and causes. Overall, 25.0% (5/20) of patients that received balloon tibioplasty reported pain during follow-up [21, 28]. Cuzzocrea et al. [21] reported a single case of occasional pain in 16.7% (1/6) of patients that received balloon tibioplasty at 6-month follow-up. This case was due to hardware-induced (long screw) osteolysis at the head of the fibula, which was alleviated with surgical removal of the hardware. Doria et al. [28] reported pain in 28.6% (4/14) of patients receiving balloon tibioplasty at 1-year follow-up, of which 1 patient experienced throbbing pain with certain positions and 3 patients experienced occasional pain. In the Doria et al. group that received conventional reduction with a metal tamp from the same study, 42.9% (6/14) of patients reported pain at 1-year follow-up, with 2 patients reporting constant pain after activity, 2 patients reporting throbbing pain with certain positions, and 2 patients reporting occasional pain. Dall’Oca et al. reported pain scores collected as a part of the Rasmussen score following arthroscopic reduction internal fixation (ARIF) and ORIF (mean follow-up approximately 2 and 6 years, respectively). Average pain for type III patients that received ARIF and ORIF was 5.69 and 5.44, respectively, with a score of 5 associated with occasional pain and a score of 6 associated with no pain [23]. The remaining studies did not provide pain data by fracture type [19, 20, 22, 24–27].

## Discussion

This study examined postoperative patient outcomes of patients with Schatzker type III tibial plateau fractures treated with a balloon tibioplasty reduction technique compared to patients treated with conventional osteosynthesis techniques. Positive patient-reported functional outcomes were observed in two studies assessing balloon tibioplasty 6-months to 1-year post-operation. Functional results and follow-up times varied in studies reporting conventional osteosynthesis. Generally, lower functional scores were observed with conventional osteosynthesis techniques at early follow-up time points (6-months and 1-year) as compared to balloon tibioplasty, although sample sizes were extremely limited and heterogeneous; additionally, functional scores were similar between both techniques at extended follow-up time points (> 2 years). This preliminary evidence suggests that balloon tibioplasty may lead to good functional outcomes for type III fractures at short-term follow-up, and that in the long term, both reduction techniques appear to result in satisfactory outcomes; however, inconsistency in study design and study quality make it difficult to draw conclusions.

Importantly, the results of this review must be interpreted in light of substantial heterogeneity around key indicators of functional outcome. The Rasmussen score was the most common functional outcome reported, yet it was only reported in less than three quarters of included studies; KSS was the second most common outcome reported, and it was only included in two studies. The maximum mean Rasmussen score (28.9) was seen with balloon tibioplasty, while the lowest (18.0) was seen in a study utilizing conventional ORIF with plate support; however, the limited number of included studies and the small size of those included complicate the ability to draw conclusions. Moreover, even the lowest reported Rasmussen score of 18.0 indicates that patients achieved at least 50% functionality. Regardless of the surgical technique utilized, the majority of patients appear to have positive outcomes at short-term and long-term follow-up.

Successful rehabilitation is critical to the success of any orthopedic procedure, with regaining ROM as a main goal of rehabilitation. Doria et al. [28] demonstrated that patients had attained full ROM (≥ 140°) and were symptom-free at 3 months. Cuzzocrea et al. [21] demonstrated that patients achieved full ROM and were weight-bearing after just 2 weeks; interestingly, they initiated rehabilitation the first day after surgery. A range of rehabilitation strategies were reported by included studies, but they generally involved limited motion and no weight-bearing for the first 6 weeks of recovery, and then partial- or full-weight-bearing as tolerated through 9–12 weeks after recovery. Details were scarce, though; others mentioned that they had a flexible rehabilitation protocol depending on the injury and surgical details [22]. The lowest mean knee ROM in the flexion plane achieved was 90°, which occurred in a study utilizing conventional ORIF with plating or cannulated screws; on the contrary, multiple studies across the two surgical technique groups achieved maximal knee flexion (≥ 140°). Additional research is needed to assess the potential advantages of each technique; however, this evidence suggests that maximal knee flexion and good ROM can be achieved through either surgical technique.

Patients’ pain levels were not consistently addressed across studies. The only study to directly compare pain levels following surgery was conducted by Doria et al. [28], which reported a lower proportion of patients with pain at 1-year follow-up, as measured with the Rasmussen scale. Fortunately, intervention may improve patient pain, as evidenced by one patient who had hardware-induced pain that was then relieved following hardware removal [21]. Dall’Oca et al. [23] also reported pain data; however, these data were obtained over 9 years and are difficult to compare to the other studies due to disparity in follow-up time length. Overall, no definitive conclusions could be drawn about short- or long-term pain levels in relation to surgical technique due to the inconsistency of reporting and scarcity of data points.

Few studies utilized imaging to examine the success of reduction techniques at follow-up time points < 1 year. Doria et al. [28] described surgical success with balloon tibioplasty on the first day after operation, with no residual depression observed across 75% of the joint area on CT. Similarly, Cuzzocrea et al. [21] described early success at 6 months following balloon tibioplasty; CT scan performed on post-operative day 1 revealed recovery over 70% of the joint area of interest free of articular bony fragments. Additionally, at 6 months, no signs of subsidence or screw mobilization were seen. In conventional osteosynthesis studies, imaging regimes varied in terms of frequency, modality (X-ray, CT, etc.), and utilization of imaging; for example, some measured average distance of bony collapse [25], some measured average weeks to bony union [22], and still others used the Rasmussen radiological assessment, which is scored based on osseous depression, condylar widening, angulation (varus/valgus), and other domains [23]. More consistent reporting and uniform imaging regimens are needed in order to draw conclusions around these outcomes.

While there are relative advantages and disadvantages of each surgical approach for tibial plateau fracture, many who have done research on the newer balloon-assisted technique have asserted its superiority and advocate for wider use among surgeons. Based on their in vitro evaluation of the techniques, Broome et al. [12] described the balloon-assisted advantage of creating a contained and symmetrical defect for additional subchondral support; similarly, in their clinical experience, Doria et al. [28] observed that balloon tibioplasty increased the area of force transmission and facilitated fracture reduction with minimal trauma to surrounding areas. By contrast, complications associated with ORIF include muscular and soft tissue damage, damage to the articular surface, surgical site infection, postoperative pain/arthritis, and scarring [29–31]; these risks are heightened in the elderly population, who experience tibial plateau fractures at the highest rates [1] and are more likely to have predisposing comorbidities such as osteoporosis and diabetes [32]. Moreover, a 2017 study of 75 patients by Elabjer et al. [30] found that arthroscopic techniques for tibial plateau fracture reduction have a shorter hospital stay by as much as 2 days (3.10 ± 0.63 vs. 5.51 ± 1.66; *p* = 0.0001). For all these reasons, balloon tibioplasty deserves wider consideration among surgeons treating tibial plateau fractures.

Nevertheless, other aspects of balloon tibioplasty should be noted here, including potentially higher cost to patients or institutions [33]. Additionally, balloon tibioplasty may not be appropriate for all patients; for example, Mauffrey et al. note that a complete cortical ring is necessary to achieve articular reduction and to avoid the “trap-door effect,” leading to insufficient reduction [8]. Finally, balloon tibioplasty is not without its own risks or complications, including burst balloon, cement extrusion, and failed reduction of the depressed articular fragment [34]. Research indicates that these complications are infrequent, but more data are needed from direct comparison studies to evaluate the relative merits and ideal populations for each surgical technique.

### Limitations

The main limitation of this study was low data availability around balloon tibioplasty, and even lower availability of those specifically describing outcomes from patients with type III fractures; as such, it was not possible to have more stringent criteria around study quality for inclusion in this review. Additional studies designed to mitigate potential bias are needed. Surgical protocols (including rehabilitation) and reporting of outcomes were extremely heterogeneous across all studies, which made it difficult to comprehensively assess differences arising from surgical techniques. With such significant potential confounding factors at play in this preliminary set of evidence, no strong conclusions may be drawn. Further heterogeneity in follow-up times and overall patient numbers must be taken into consideration when interpreting these results. Future studies with consistent reporting of surgical technique and patient-reported outcomes are needed to allow for direct comparisons of different treatment modalities.

## Conclusion

In a small number of studies reporting balloon tibioplasty to reduce articular depression of a lateral tibial plateau fracture, promising outcomes were observed in terms of patient pain, ROM, and overall functioning. Reduction of a lateral tibial plateau depression requires careful technique so as not to cause iatrogenic trauma to the area, and early studies have shown favorable results utilizing a soft kyphoplasty balloon for elevation. Further research is needed to better characterize patient outcomes, particularly long-term outcomes following balloon tibioplasty versus conventional osteosynthesis techniques in patients with a Schatzker type III fracture.

## Supplementary Information

**Additional file 1.** Risk of bias assessment of included studies.

## Abbreviations

CT: Computed tomography
3D: Three-dimensional reconstruction
KSS: Knee Society Score
JBI: Joanna Briggs Institute
ORIF: Open reduction and internal fixation
ROM: Range of motion
ARIF: Arthroscopic reduction internal fixation
